# Effect of Seasonal Variation on the Relationship of Indoor Air Particulate Matter with Measures of Obesity and Blood Pressure in Children

**DOI:** 10.5696/2156-9614-11.30.210610

**Published:** 2021-05-28

**Authors:** Anye Chungag, Godwill Azeh Engwa, Constance Rufaro Sewani-Rusike, Benedicta Ngwenchi Nkeh-Chungag

**Affiliations:** 1 Department of Geography and Environmental Sciences, Faculty of Science and Agriculture, University of Fort Hare, Alice, South Africa; 2 Department of Biological and Environmental Sciences, Faculty of Natural Sciences, Walter Sisulu University, Mthatha, South Africa; 3 Department of Human Biology, Faculty of Health Sciences, Walter Sisulu University, Mthatha, South Africa

**Keywords:** particulate matter, obesity, high blood pressure, indoor air pollution, seasonal variation

## Abstract

**Background.:**

Particulate matter (PM) air pollution is an important environmental health risk factor. Although some studies have shown PM to be associated with obesity and hypertension, very few studies have assessed the association of indoor PM specifically with obesity and blood pressure measures in children with respect to seasonal variation.

**Objectives.:**

The present study investigated the relationship of PM with obesity and blood pressure variables in children across the winter and summer seasons.

**Methods.:**

A comparative descriptive approach was adopted and school children from 10–14 years of age from selected rural and urban localities of the Eastern Cape Province of South Africa were assessed in winter and summer. Anthropometric measurements were taken, including height, weight, waist circumference, body mass index (BMI), and total fat mass (TFM), while blood pressure variables including systolic blood pressure (SBP), diastolic blood pressure (DBP) and heart rate (HR) were measured. Indoor air PM concentrations were measured in the classrooms in the presence of children.

**Results.:**

The prevalence of obesity and hypertension in children were 13.4% and 5.1% in winter and 12.9% and 1.0% in summer, respectively. High blood pressure was more prevalent in children in rural areas, while the prevalence of obesity in children was higher in urban areas. Particulate matter was significantly (p<0.05) higher in rural areas compared to urban areas. Obese children in summer had a greater than 3-fold association (AOR: 3.681, p=0.005) with 4th interquartile range (IQR) of PM5 and a greater than 3- and 4-fold association (AOR: 3.08; 4.407; p<0.05) with 2nd and 4th IQR of PM10, respectively, than their overweight, normal weight or underweight counterparts. High blood pressure was not associated (p< 0.05) with PM.

**Conclusions.:**

High concentrations of indoor PM were positively associated with obesity in children in summer, particularly among rural children. This association could be accounted for by location and seasonal differences.

**Participant Consent.:**

Obtained

**Ethics Approval.:**

Ethics approval was obtained from the Health Sciences Ethics Committee of Walter Sisulu University, South Africa (Ref No: CHI011SCHU01).

**Competing Interests.:**

The authors declare no competing financial interests.

## Introduction

Epidemiological studies show that air pollution greatly contributes to morbidity and mortality.^1^,^2^

Air pollution is ranked ninth among modifiable risk factors for diseases and was responsible for over 3 million deaths globally in 2010.^3^ Air pollution has also been found to contribute to respiratory and cardiovascular diseases.^4^,^5^,^6^ Air pollution usually results from combustion or other environmental activities resulting in the release of gases such as carbon monoxide (CO), ground-level ozone (O_3_), nitrogen dioxide (NO_2_), sulphur dioxide (SO_2_), etc., as well as particulate matter (PM).^7^,^8^ Exposure to air pollutants such as PM, SO_2_, NO_2_ and volatile organic compounds (VOCs) has been associated with obesity, hypertension and various respiratory and cardiovascular diseases.^4^,^9^,^10^ Among these air pollutants, most adverse health effects have been attributed to PM, especially the particulate fractions sized between PM_2.5_ to PM_10_.^11^ Particular matter refers to small tiny solid particles and liquid droplets in the atmosphere with sizes in micrometers (μm), which include dust, dirt, soot, smoke and drops of liquids that can cause air pollution. Course (bigger) particulate matter, called PM_10_, is 10 μm in diameter while fine (smaller) particulate matter, called PM_2.5_ is 2.5 μm. PM_2.5_ is more dangerous because it can get into deep parts of the lungs and even penetrate into circulation.^11^ Most of the findings on the health effects of small PM have stemmed from outdoor pollution studies.^12^,^13^ According to the World Health Organization (WHO), outdoor air pollution accounts for about 2 million deaths globally every year, with the majority of these deaths occurring in low- and middle-income countries (LMIC).^14^ Although attention has been primarily on outdoor air pollution, the current trend is to look at the health impact of indoor air pollution which is increasingly been linked with ill health.

Indoor air pollution results mainly from the infiltration of the indoor environment by harmful outdoor chemicals and household pollutants. There is increasing evidence that indoor air pollution could be about 10 times worse than outdoor air pollution^15^ since confined environments such as houses could promote the build-up of potential pollutants more than open spaces. There is increasing evidence that indoor air pollution may have a greater impact on health as debilitating aerosols infiltrate buildings where most of the exposure typically takes place.^16^ It has been suggested that indoor air pollution is more of a health concern in LMIC than outdoor air pollution since the use of solid fuel materials for combustion is more common in LMIC.^15^ Poor ventilation of buildings results in continuous recycling of confined indoor air which becomes a health hazard to occupants; especially in young children and the elderly who spend more than 90% of their time indoors.^17^ Reports show that particulate matter pollution accounts for over 16% of indoor air pollution from solid fuels. Moreover, solid fuel indoor air pollution accounted for 3.5 million deaths and about 4.5% global daily-adjusted life years (DALYs) in 2010.^18^

**Abbreviations**
*DBP*: Diastolic blood pressure
*LMIC*: Low- and middle-income countries
*SBP*: Systolic blood pressure

A few studies have shown PM to be associated with obesity as well as hypertension.^19^,^20^ However, the majority of these studies have focused primarily on indoor air pollution in adults.^20^ The relationship between indoor air PM and obesity/hypertension in children has received little attention in Africa. In South Africa, the prevalence of childhood obesity and hypertension is on the rise.^21^ Reports from the Eastern Cape region show an increasing prevalence of obesity and hypertension with increased risk of cardiovascular diseases.^21^,^22^ The rapid urbanization of most sub-Saharan African communities accompanied by lifestyle changes may influence health differently in urban and rural settings. Furthermore, seasonal variations may affect the availability and distribution of PM as was previously suggested by Kurai and colleagues.^23^ Thus, the present study aimed to assess the effect of seasonal variation on the relationship of indoor PM with obesity and blood pressure measures in children selected from rural and urban schools of the Eastern Cape Province of South Africa.

## Methods

A comparative descriptive approach was employed to assess the relationship of PM with obesity and blood pressure measures in winter and summer. Children 10–14 years of age were recruited into the study from three urban and four rural middle schools of the Eastern Cape Province of South Africa. A total of 540 children were recruited in winter and 411 children continued the study in summer, yielding a response rate of 76.11%. Their anthropometric and blood pressure measurements were taken in winter 2016 and assessed again in summer 2017, six months after the initial measurements. All the schools included in this study were public schools with a meal program which ensures that children at the schools eat the same meals daily five days a week. We also ensured that the children had comparable physical activity.

### Study area

**Figure 1:**
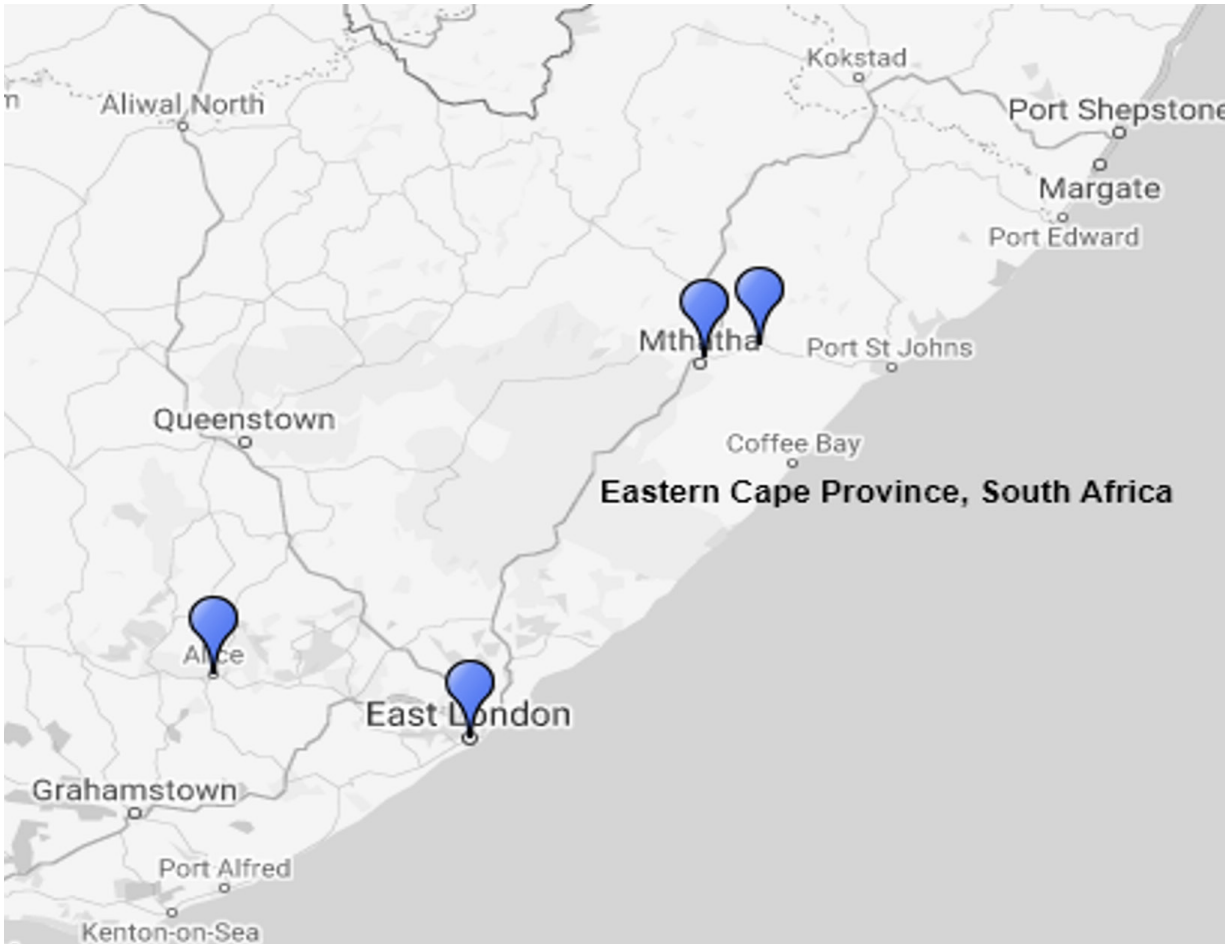
Urban and rural areas of the Eastern Cape Province of South Africa. Marked areas indicate the study sites.

This study was conducted in East London and Mthatha, which are urban areas, and in Alice and Libode, rural areas of the Eastern Cape Province of South Africa with geographical coordinates 32.2968º S and 26.4194º E *(Figure 1).* Summer, which occurs from mid-October to mid-February, and winter which occurs between May and July, were the seasons chosen for the study because they are distinct weather conditions that have serious environmental impact. Winter is usually cold and windy, while summer is hot and rainy in most parts of the country. Autumn and spring are intermediary weather conditions that transition between summer and winter, and between winter and summer, respectively. Hence, the focus of this study was the seasonal variation between winter and summer which are very distinct seasons in the Eastern Cape Province of South Africa. The Eastern Cape Province is one of the poorest regions in South Africa and is mostly dominated by rural areas and populations. Most of the rural areas including Libode, Qumbu, Tsolo, Qanda, etc., have untarred roads and make use of solid fuel for cooking, while the urban areas such as Mthatha, East London, Port Elisabeth, Queenstown etc. are well-developed regions with tarred roads.

### Ethics approval

The study was conducted in accordance with the guidelines of the Helsinki Declaration (reviewed version of 2008)^24^ as well as local and national regulations in South Africa.^25^ Ethical approval was obtained from the Health Sciences Ethics Committee of Walter Sisulu University, South Africa (Ref No: CHI011SCHU01). After explanation of the study objectives and methods, we obtained written informed consent from the relevant school authorities, and parents or legal guardians of participants. Participation was voluntary. The study adhered to the standards of reporting and in accordance with the National Data Protection Act, each participant was assigned a code, and data/samples were stored anonymously. There were no important changes to the methods after study commencement.

### Inclusion/exclusion criteria

Male or female children aged 10–14 years who were free from any renal, pulmonary and chronic cardiovascular diseases were recruited for the study. Ill and physically challenged individuals as well as individuals on blood pressure lowering medication were excluded from the study.

### Determination of particulate matter count

Indoor air from 23 classrooms was sampled. Particulate matter count was measured with the aid of a handheld particle counter (Met-One-Model-804, Met One lnstruments, lnc., Washington, USA) which was placed at a height of 60 cm above the floor in the middle of each classroom. Measurements were carried out within a period of 15 minutes and performed twice between 8am–1pm when students were in the classrooms. The average of the two readings was recorded. Measurements were taken once in winter and once in summer for each classroom. The results were expressed as μg/m^3^.

### Anthropometric measurements

Anthropometric measurements were performed in accordance with WHO guidelines.^26^ Participants were requested to stand upright with feet together with arms hanging freely. Waist circumference and hip circumference were measured with a non-stretch tape in centimeters (cm) and the waist to hip ratio (WHR) was calculated. Height was measured using a stadiometer. Participants were requested to take off their shoes and to step on the stadiometer platform, close to the stadiometer rod. The movable bar was lowered to just touch the head of the participant and the reading was made to the nearest centimeter. An Omron body composition monitor (BR511) was used to measure the weight, total fat mass (TFM) and total muscle mass (TMM) of the participants and personal data such as height, age and sex were entered into the monitor to determine the body mass index (BMI). The BMI was converted to percentiles for age and sex according to the International Obesity Task Force (IOTF) criteria.^27^ Underweight was considered at <5^th^ percentile, normal weight: ≥5^th^ to <85^th^ percentile, overweight: ≥ 85^th^ to <95^th^ percentile and obese: ≥95^th^ percentile.

### Blood pressure measurements

Participants were required to rest in the seated position for ten minutes after which their right upper arm was fitted with an appropriate arm size cuff and blood pressure (BP) measured at three-minute intervals using the Omron (Hem 7120) automated blood pressure machine. The mean of three recordings of systolic blood pressure (SBP), diastolic blood pressure (DBP) and heart rate (HR) were computed. The average of the BP readings was determined and converted to BP percentiles for sex, age and height and classified according to the American Academy of Pediatrics (AAP) 2017 guideline as normotensive: SBP and/or DBP < 90^th^ percentile; elevated BP: SBP and/or DBP > 90^th^ < 95^th^ percentile or high BP: SBP and/or DBP ≥ 95^th^ percentile.^28^

### Statistical analysis

Data were analyzed using IBM Statistical Package for Social Sciences (SPSS) Version 20. Data were presented as mean± standard deviation (SD). Two-way analysis of variance (ANOVA) was used to determine mean differences of particulate matter based on particle size and location as well as mean differences of anthropometric and blood pressure measures based on location and sex. Multinomial logistic regression was employed to assess the relationship of particulate matter (independent variable) with obesity and high blood pressure, being the dependent variables. Age, sex (male/female) and location (urban/rural) were entered into the model to adjust for confounding variables and obtain the adjusted odd ratio (AOR). Differences with a *p*-value ≤ 0.05 were considered significant.

## Results

**Figure 2:**
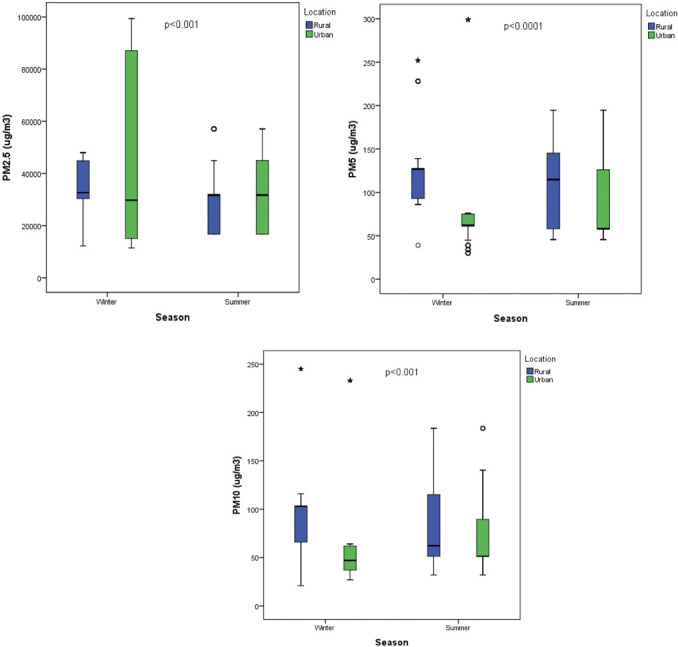
Particulate matter counts in rural and urban classrooms during winter and summer. A p-value ≤ 0.05 is considered to be significant.

Particulate matter (μg/m^3^) was assessed in 11 urban and 12 rural classrooms from which children were recruited for the study. Classroom indoor PM count varied significantly (*p*<0.05) between winter and summer and between rural and urban classrooms. In general, levels of PM_5_ and PM_10_ were higher in rural classrooms compared to urban classrooms in both winter and summer, except for PM_2.5_ which was higher in rural classrooms in winter while in summer, it was higher in urban classrooms *(Figure 2).*

### Characteristics of study population

The age, height, waist circumference, WHR, BMI, TFM, SBP and DBP of the children were similar (*p*>0.05) in winter and summer. However, the weight, hip circumference, TMM and HR of the children were different (*p*<0.05) in winter and summer (*Table 1*). The prevalence of obesity in winter (13.4%) was comparable to the prevalence in summer (12.9%), while the prevalence of high blood pressure was higher in winter (5.1%) than in summer (1.0%). The prevalence of overweight was lower in winter (9.3%) than in summer (12.2%), while elevated blood pressure was higher in winter (32.8%) and summer (20.9%). The prevalence of obesity was generally higher in urban areas while high blood pressure was generally higher in rural areas *(Table 2).*

**Table 1:** Characteristics of Study Participants

	**Winter**				**Summer**				

	**Rural (190)**		**Urban (221)**		**Rural (190)**		**Urban (221)**		**p-value**
	**Females**	**Males**	**Females**	**Males**	**Females**	**Males**	**Females**	**Males**	
N (%)	102	88	121	100	102	88	121	100	
Age	11.71±1.01	11.69±0.86	11.44±0.91	11.63±0.97	12.11±0.89	12.22±1.03	12.24±0.99	12.29±1.04	0.171
Height	149.07±8.11	144.08±20.29	146.46±21.56	143.93±19.60	154.15±7.72	153.72±8.56	152.02±8.21	149.58±8.51	0.455
Weight	44.28±12.14	39.89±12.14	46.86±18.22	45.81±22.23	50.04±15.25	46.02±10.55	46.77±13.56	45.21±13.26	0.044
BMI	19.83±4.34	18.58±4.27	19.98±4.65	19.48±4.03	20.94±5.85	19.28±3.93	19.94±4.61	19.99±4.77	0.630
WC	67.29±8.73	63.46±9.67	68.91±1 1.21	67.06±9.97	69.94±12.14	67.60±8.19	69.49±10.34	69.27±10.78	0.436
HC	84.43±10.47	77.60±11.61	85.04±12.08	82.44±9.87	85.75±13.84	84.85±10.63	85.27±11.56	85.01±11.53	0.036
WHR	0.79±0.04	0.82±0.09	0.81±0.06	0.81±0.07	0.83±0.25	4.79±0.05	0.82±0.09	0.81±0.09	0.121
TFM	22.97±8.89	18.56±8.08	23.75±9.51	21.18±8.94	25.22±31.15	20.27±9.95	23.09±9.51	23.78±9.43	0.639
TMM	33.13±2.69	35.47±3.21	32.53±2.76	34.24±4.03	34.35±5.11	35.19±4.33	34.18±8.56	32.87±3.38	0.003
SBP	114.78±9.50	111.54±10.28	110.82±10.35	110.03±9.94	106.82±10.58	106.09±12.25	102.63±10.35	102.56±8.24	0.608
DBP	74.28±7.32	71.26±6.44	72.14±7.46	70.56±7.08	73.83±22.42	71.97±8.06	70.41±8.19	70.94±6.37	0.680
HR	89.44±11.75	86.13±12.06	89.18±12.06	83.11±12.26	80.21±11.34	80.58±12.70	81.74±13.42	80.80±11.59	0.025

Note: Results are expressed as mean±SD. Abbreviations: SD= standard deviation; N = number of participants; WC: waist circumference, BMI: body mass index, HC: hip circumference, WHR: waist to hip circumference, TFM: total fat mass, TMM: total muscle mass, SBP: systolic blood pressure, DBP: diastolic blood pressure, HR: heart rate.

**Table 2:** Prevalence of Obesity and Elevated Blood Pressure

	**Winter**				**Summer**		

		**Cohort**	**Rural (%)**	**Urban (%)**	**Cohort**	**Rural (%)**	**Urban (%)**
Obesity							
	Lean	318 (77.4)	157 (82.6)	161 (72.9)	308 (74.9)	141 (78.3)	167 (72.3)
	OW	38 (9.3)	19 (10.0)	19 (8.6)	50 (12.2)	17 (9.4)	33 (14.3)
	Obese	55 (13.4)	14 (7.4)	41 (18.6)	53 (12.9)	22 (12.2)	31 (13.4)
BP							
	Normal	255 (62.0)	105 (55.3)	150 (67.9)	321 (78.1)	132 (73.3)	189 (81.8)
	EBP	135 (32.8)	73 (38.4)	62 (28.1)	86 (20.9)	45 (25.0)	41 (17.7)
	HBP	21 (5.1)	12 (6.3)	9 (4.1)	4 (1.0)	3 (1.7)	1 (0.4)

Abbreviations: OW: overweight, BP: blood pressure, EBP: elevated blood pressure, HBP: high blood pressure

### Relationship of particulate matter with obesity and blood pressure measures

Multinomial logistic regression analyses, after adjusting for age, sex and location showed no association (*p*>0.05) between PM and high blood pressure in children both in winter and summer. Moreover, regression analysis showed obesity to be associated with PM_5_ and PM_10_ in summer only after adjusting for age, sex and location as obesity was positively associated (*p*<0.05) with the interquartile range (IQR) of PM in summer and not in winter. Obese children in summer had a 3.7-times greater association (AOR: 3.681, *p*=0.005) with 4^th^ IQR of PM_5_ than their overweight, normal weight or underweight counterparts. In addition, obese children in summer had a 3- and 4-times greater association with 2^nd^ IQR (AOR: 3.08; *p*<0.05) and 4^th^ IQR (AOR: 4.407; *p*<0.05) of PM_10_ than their overweight, normal weight or underweight counterparts, respectively *(Table 3).* Further regression analysis after adjusting for age and sex to assess the relationship between obesity and PM based on location showed that there was an association between obesity and PM in rural children in summer. Obese children in summer had a 6-times greater association (AOR: 6.172, *p*<0.05) with 4^th^ IQR of PM_5_ and a 9-times greater association with 2^nd^ IQR (AOR: 9.54; *p*<0.05) and 4^th^ IQR (AOR: 11.47; *p*<0.05) of PM_10_ than their overweight, normal weight or underweight counterparts (*Table 4*). Furthermore, multinomial logistic regression analyses, after adjusting for season, showed an association of 4^th^ IQR of PM_5_ (AOR: 0.459, *p*<0.05) and PM_10_ (AOR: 0.459; *p*<0.05) with obesity (*Table 5*).

**Table 3:** Regression Analysis of Particulate Matter with Obesity in Summer and Winter

	**Winter**				**Summer**				

	**IQR**	**β**	**AOR (95% CI)**	**p-value**		**IQR**	**β**	**AOR (95% Cl)**	**p-value**
PM_2.5_	1^st^		Ref		PM_2.5_	1^st^		Ref	
	2^nd^	0.581	1.787 (0.66–4.81)	0.250		2^nd^	−0.346	0.707 (0.32–1.56)	0.390
	3^rd^	−0.914	0.401 (0.12–1.32)	0.132		3^rd^	0.488	1.629 (0.74–3.57)	0.222
	4^th^	0.383	1.467 (0.67–3.21)	0.337		4^th^	−0.503	0.605 (0.25–1.46)	0.266
PM_5_	1^st^		Ref		PM_5_	1^st^		Ref	
	2^nd^	−0.388	0.679 (0.31–1.48)	0.331		2^nd^	0.783	2.187 (0.875–5.46)	0.094
	3^rd^	−0.958	0.384 (0.09–1.49)	0.165		3^rd^	0.571	1.770 (0.67–4.66)	0.248
	4^th^	−0.452	0.636 (0.24–1.69)	0.366		4^th^	1.303	3.681 (1.49–9.061)	**0.005**
PM_10_	1^st^		Ref		PM_10_	1^st^		Ref	
	2^nd^	−0.605	0.546 (0.25–1.18)	0.123		2^nd^	1.125	3.080 (1.07–8.87)	**0.037**
	3^rd^	−0.922	0.398 (0.112–1.408)	0.153		3^rd^	0.861	2.365 (0.78–7.17)	0.129
	4^th^	−0.254	0.776 (0.31–1.95)	0.589		4^th^	1.483	4.407 (1.56–12.44))	**0.005**

Abbreviations: PM: particulate matter, IQR: interquartile range, AOR: adjusted odd ratio, CI: confidence interval, β: regression coefficient, Ref: reference group.

**Bold** indicates significance

**Table 4:** Regression Analysis of Particulate Matter with Obesity in Summer and Winter by Location

	**Winter**						**Summer**					

	**Rural**			**Urban**			**Rural**			**Urban**		
	IQR	β	AOR	IQR	β	AOR	IQR	β	AOR	IQR	β	AOR
PM_2.5_	1^st^	Ref		1^st^	Ref		1^st^	Ref		1^st^	Ref	
	2^nd^	1.314	3.721*	2^nd^	0.428	1.534	2^nd^	−0.810	0.445	2^nd^	−0.031	0.969
	3^rd^	——	——	3^rd^	−1.740	0.175	3^rd^	0.218	1.243	3^rd^	0.759	2.136
	4^th^	——	——	4^th^	0.521	1.683	4^th^	−2.049	0.129	4^th^	0.070	1.073
PM_5_	1^st^	Ref		1^st^	Ref		1^st^	Ref		1^st^	Ref	
	2^nd^	1.125	3.081	2^nd^	−0.775	0.461	2^nd^	1.820	6.172*	2^nd^	0.172	1.188
	3^rd^	——	——	3^rd^	——	——	3^rd^	0.774	2.169	3^rd^	0.561	1.752
	4^th^	−0.867	0.420	4^th^	0.026	1.027	4^th^	2.072	7.940*	4^th^	.943	2.569
PM_10_	1^st^	Ref		1^st^	Ref		1^st^	Ref		1^st^	Ref	
	2^nd^	17.165	28.48	2^nd^	−1.251	0.861**	2^nd^	2.256	9.540*	2^nd^	0.608	1.837
	3^rd^	16.014	90.07	3^rd^	——	——	3^rd^	1.254	3.505	3^rd^	0.862	2.369
	4^th^	15.545	56.38	4^th^	−.150	0.286	4^th^	2.440	11.473*	4^th^	1.189	3.285

Note: ^*^indicates significant association where *p<*0.05;

^**^indicates significant association where *p<*0.01.

Abbreviations: PM: particulate matter, IQR: interquartile range, AOR: adjusted odd ratio, CI: confidence interval, β: regression coefficient, Ref: reference group

**Table 5:** Regression Analysis of Particulate Matter with Obesity

	**IQR**	**β**	**AOR (95% CI)**	***p*-value**
PM_2.5_	1^st^		Ref	
	2^nd^	−0.016	0.984 (0.57–1.71)	0.956
	3^rd^	−0.248	0.407 (0.43–1.40)	0.407
	4^th^	−0.567	0.67 (0.29–1.08)	0.083
PM_5_	1^st^		Ref	
	2^nd^	−0.150	0.861 (0.49–1.50)	0.598
	3^rd^	−0.228	0.796 (0.45–1.41)	0.434
	4th	−0.778	0.459 (0.24–0.88)	**0.019**
PM_10_	1^st^		Ref	
	2^nd^	−0.264	0.768 (0.44–1.35)	0.358
	3^rd^	−0.354	0.702 (0.39–1.25)	0.228
	4^th^	−0.869	0.419 (0.22–0.81)	**0.009**

**Bold** indicates significance

## Discussion

The present study investigated indoor air quality in selected classrooms in the Eastern Cape Province of South Africa and its relationship with anthropometric and blood pressure measures in winter and summer. Particulate matter counts varied with seasons and rural versus urban location of schools. Our findings showed seasonal variation in fine particle distribution in classrooms when students were in classrooms. Particulate matter was more present in winter than in summer and relatively higher levels were observed in rural areas. This finding concurs with that of previous reports which found that seasonal variation as well as human activities affect the distribution of PM.^7^,^23^ Particulate matter is known to have health implications and is considered to be a modifiable risk factor for hypertension, respiratory and cardiovascular diseases.^4^–^6^

High blood pressure and obesity amongst other factors are major risk factors for cardiovascular diseases.^29^,^30^ Childhood obesity and high blood pressure are steadily on the rise in LMIC in both urban and rural communities.^31^,^32^ Previous studies in the Eastern Cape Province of South Africa have shown a high prevalence of high blood pressure and obesity in adolescents.^21^,^22^ Findings in this study showed low prevalence of obesity and high blood pressure although a high prevalence of elevated blood pressure was observed.

High blood pressure as well as obesity, respiratory disorders,^33^,^34^ cardiovascular diseases,^35^,^36^ lung cancer^37^,^38^ etc. in children have recently been associated with PM. Particulate matter air pollution is one of the world's largest single environmental health risk factors.^39^ Airborne PM, especially particles with diameters less than 10 μm are of serious health concerns as they are increasingly shown to contribute to mortality and morbidity.^1^,^2^ Due to its small size, PM_10_ is capable of accessing the vascular system through the thin respiratory membrane. Once in the vascular system, PM_10_ can cause cardiovascular diseases.^40^

Since cardiovascular risk factors such as obesity and hypertension in children tracks into adulthood, it is important that the impact of air pollution in childhood be addressed to reduce the risk of developing cardiovascular diseases in adulthood. Several studies have investigated the relationship between air traffic pollution and obesity and/or high blood pressure.^19^,^41^,^42^ The results have so far not been conclusive.^19^,^41^,^43^ One of the reasons why varying results may be obtained could be explained by seasonal variations in the concentration and distribution of pollutants such as PM across different locations.^23^ Findings of this study showed no association between high blood pressure and particulate matter in children both in winter and summer. Our finding is in contrast to a study in China where long-term exposure to ambient PM air pollution was associated with increased blood pressure and higher prevalence of hypertension in children and adolescents.^44^ Although a clear biological mechanism to explain the link between PM air pollution and blood pressure has not been fully established, there have been a few hypotheses. It has been proposed that PM exposure can induce oxidative stress^45^ as well as systemic inflammation which can affect vascular functions and hemodynamic responses.^46^,^47^ It has also been suggested that inhalation of PM can cause changes in the autonomic nervous system,^48^ particularly favoring the sympathetic nervous system to mediate arterial vasoconstriction. There is also a possibility that PM can penetrate the alveolar capillary membrane and be transported into circulation where it can directly affect blood vessels.^49^ Moreover, it has been hypothesized that air pollution and obesity may synergistically have an effect on blood pressure since both can induce systemic inflammation,^50^ a factor that is critical in the development of hypertension.^46^

Since obesity is generally known as one of the major risk factors of hypertension and cardiovascular diseases, the present study investigated the relationship between obesity and PM. Our findings showed higher IQR of PM_5_ and PM_10_ to be associated with obesity in summer after adjusting for age, sex and location. This finding agrees with a previous study in China which showed an increased prevalence of obesity with an IQR increase in PM_10_.^51^ A systemic review and meta-analysis study has also shown exposure to ambient PM to be positively associated with childhood obesity.^52^ Another study showed that high levels of PM_2.5_ were shown to have a minor effect on obesity and contributed to an increase in the prevalence of type 2 diabetes in the United States of America.^43^ Although studies have shown an association between PM and obesity, the causation remains unclear. However, a study suggested that air pollutants such as PM are able to infiltrate and activate immune-competent cells like macrophages and monocytes leading to adipocyte hypertrophy.^53^ It has been reported that PM_2.5_ exposure may increase the diameter of adipocytes via the activation of NADPH oxidase-derived superoxide, leading to obesity.^54^

A systemic review demonstrated that associations between air pollution and body weight status varied with sex, age group, and type of air pollutant.^55^ After controlling for age, sex and location, our study showed a relationship between obesity and PM in summer and not in winter. In particular, after adjusting for season, higher IQRs of PM_5_ and PM_10_ were associated with obesity. Our findings suggest that seasonal differences may be accountable for the relationship between obesity and PM. This finding is supported by a previous study which investigated the effects of spring and winter PM on airway inflammation and allergies in a mouse asthma model and observed seasonal variations in the effects of PM on asthma-related airway inflammation.^23^ In addition, the present study showed that the relationship between obesity and PM in summer was more prominent in rural children as obesity in rural children had a 6-fold greater association with increased IQR of PM_5_ and a 9-fold greater association with increased IQR of PM_10_ than their overweight, normal weight or underweight counterparts in summer. This may suggest that more PM is present or generated in rural areas than in urban areas, as supported by our findings. This implies that apart from seasonal variation, location may affect the distribution of PM and influence its relationship with risk of metabolic disease. It may be suggested that most PM observed in the present study is a product of solid fuel combustion,^7^ an activity that differs between urban and rural settings as high level of PM in rural areas may have originated from dust produced by untarred roads and/or combustion of solid fuels such as wood for cooking. However, the source of PM in urban areas during summer remains unclear. This study supports previous studies which have shown human activities and climate change to affect the distribution and increase the level of fine PM.^56^ However, this study may be limited in that it did not monitor the level of solid fuel used during the study period which could be the source of PM in the study area. In addition, covariates such as diet, sedentary lifestyle and level of physical activity of children which could potentially influence these results were not monitored. Therefore, these findings are preliminary which could be further investigated in more robust prospective cohort designs to adjust for more potential confounding variables.

## Conclusions

This present study showed that high concentrations of PM were positively associated with obesity in children in summer and prominent in rural children. This association of PM with obesity could be accounted for by location and seasonal differences. Because obesity is associated with increased risk of metabolic and cardiovascular diseases, concerns about the harmful effects of air pollution on children's health should be a top priority for public health policy and it should be underscored in primordial and primary prevention of chronic diseases.
